# Leaping the Boundaries in Laparoscopic Liver Surgery for Hepatocellular Carcinoma

**DOI:** 10.3390/cancers14082012

**Published:** 2022-04-15

**Authors:** Gianluca Cassese, Ho-Seong Han, Boram Lee, Hae Won Lee, Jai Young Cho, Roberto Troisi

**Affiliations:** 1 Department of HPB Surgery, Seoul National University Bundang Hospital, Seongnam 13620, Korea; gianluca.cassese@unina.it (G.C.); boramlee0827@snubh.org (B.L.); lansh@hanmail.net (H.W.L.); jycho@snubh.org (J.Y.C.); 2 Minimally Invasive and Robotic HPB Surgery Unit, Department of Clinical Medicine and Surgery, Federico II University, 80131 Naples, Italy; roberto.troisi@unina.it

**Keywords:** laparoscopic liver resection, hepatocellular carcinoma, overcoming the limits, minimally invasive liver surgery

## Abstract

**Simple Summary:**

Recent advances in surgical techniques and perioperative management lead to a redefinition of the actual frontiers of Laparoscopic Liver Resection (LLR) by including patients with more advanced disease. Nonetheless, because of both underlying liver conditions and technical difficulty, LLR for Hepatocellular Carcinoma (HCC) is still considered as a challenging procedure. Specific concerns exist about LLR in cirrhotic patients, posterosuperior segments, giant and multiple tumors, as well as repeat resections. This review focuses on the specific limits of this approach in HCC patients in order to put into practice all the pre- and intra-operative precautions to overcome their boundaries, making this technique the standard of care within high-volume hepatobiliary centers.

**Abstract:**

The minimally invasive approach for hepatocellular carcinoma (HCC) had a slower diffusion compared to other surgical fields, mainly due to inherent peculiarities regarding the risks of uncontrollable bleeding, oncological inadequacy, and the need for both laparoscopic and liver major skills. Recently, laparoscopic liver resection (LLR) has been associated with an improved postoperative course, including reduced postoperative decompensation, intraoperative blood losses, length of hospitalization, and unaltered oncological outcomes, leading to its adoption within international guidelines. However, LLR for HCC still faces several limitations, mainly linked to the impaired function of underlying parenchyma, tumor size and numbers, and difficult tumor position. The aim of this review is to highlight the state of the art and future perspectives of LLR for HCC, focusing on key points for overcoming currents limitations and pushing the boundaries in minimally invasive liver surgery (MILS).

## 1. Introduction

Liver cancer is the fifth-most-common cancer in the world and the fourth-most-common cause of cancer-related death [1]. With an estimated incidence from 500,000 to 1 million per year, hepatocellular carcinoma (HCC) accounts for about 90% of liver cancers and is still associated with a poor prognosis [2]. When it is diagnosed in the early stages, 5-year overall survival (OS) reaches 50–70%, thanks to the advances in both surgical and medical therapy [2]. Surgical treatments include liver transplantation (LT) and liver resections, with a recurrence rate as high as 20% after LT and 70% after liver resection [3]. LT is the best curative treatment in cirrhotic patients, but due to organ shortages and the long waiting times associated with the consequent risk of dropout for tumor progression, it should be reserved for patients who are not candidates for LR or RFA due to uncompensated cirrhosis, patients with bad prognostic factors on pathological examination after resection, and those with recurrent HCC in transplantable patients [4]. Accordingly, liver resection is still considered the first-line treatment for HCC in patients with compensated cirrhosis [5]. Thermal ablation is considered to be effective only for lesions smaller than 3 cm when technically feasible. On the other side, for non-resectable liver disease, trans-arterial chemoembolization (TACE) represents the treatment of choice if the patient has a suitable performance status. Medical therapy is reserved for cases with disseminated disease or when other therapies are not feasible. To date, it is mainly based on the use of sorafenib, a kinase inhibitor, but thanks to an improved understanding of molecular pathways of HCC carcinogenesis, other immunotherapy drugs are licensed in some countries or currently in an advanced phase of clinical trials [6,7].

Since the first laparoscopic liver resection (LLR) was reported by Reich and colleagues in 1991 [8], its spreading has been slower when compared to other surgical specialties. This has been due to different reasons, including the technical complexity of parenchymal transection and hilar dissection, the risk for massive bleeding, the oncological concerns about resection margins (limited by the initial unavailability of intraoperative ultrasounds), and the consideration of cirrhotic patients as too fragile and complex for a minimally invasive approach. Slowly, more and more papers focusing on LLR have been published, indicating that minimally invasive liver surgery (MILS) is a viable option for both primary and secondary liver diseases [9].

In the setting of hepatocellular carcinoma (HCC) treatment, international guidelines have officially approved the use of laparoscopy in the treatment of early-stage disease [10,11]. Indeed, different authors have proposed that MILS could decrease the risk for postoperative decompensation of HCC patients, and in everyday practice, high-volume centers routinely perform LLR also for challenging cases in fragile cirrhotic patients [12,13].

However, several limitations to the universal adoption of LLR for HCC still exist. Firstly, the minimally invasive liver surgeon must be confident with both laparoscopy and open liver surgery. Secondly, there is the need for performing more complex procedures than for other liver pathologies, which includes anatomical resections (AR), thanks to the theoretical advantage of excising the entire primary tumor along with adjacent liver parenchyma containing micro-metastases, even if survival advantages are still debated [14,15]. Finally, other challenges are represented by tumor location, tumor size, the proximity of the tumor to large vessels, and underlying liver function. All these aspects are not considered a contraindication per se but can be limiting with regard to the laparoscopic approach.

In this review, we will first summarize the current indications and limitations for LLR for HCC, and then we will focus on the strategies for overcoming the current challenges.

## 2. Current Indications and Limitations of LLR for HCC

Several advantages have been proven in patients undergoing minimally invasive surgery in other surgical fields, including reduced length of hospital stay, postoperative pain, bleedings, complication rates related to surgical incision, and improved postoperative quality of life. However, after 30 years since the first reported LLR, a randomized trial testing the efficacy and safety of a laparoscopic approach for HCC treatment still does not exist. To date, only one randomized controlled trial investigating LLR has been published, the OSLO-COMET, focused on patients with colorectal liver metastases undergoing parenchymal-sparing liver surgery, therefore including mainly atypical resections in non-cirrhotic patients, showing reduced postoperative complications and hospital stay in the laparoscopic group [16].

Even in the absence of randomized trials, recommendations for implementation and adoption of LLR in HCC were proposed by expert consensus conferences, then followed by recent guidelines based on non-randomized studies. The first international expert consensus was held in Louisville in 2008 [17]. This conference defined univocal terminology about laparoscopic procedures (pure, hand-assisted laparoscopy, and hybrid techniques). It was highlighted that major laparoscopic liver resections had been performed with safety and efficacy equaling open surgery in highly specialized centers, however underlining the potentially unsafe and rapid dissemination of such difficult procedures in the absence of structured training and renown certification.

The Southampton Consensus Guidelines (2018) can be considered the actual clinical practices guidelines, aiming to guide “the safe development and progression of laparoscopic liver surgery” [18]. These guidelines underlined that the majority of the evidence was published from surgeons experienced in both laparoscopic techniques and liver surgery, working in high-volume centers, and recognized that in expert hands, major LLR is associated with reduced hospital stay and blood loss, while oncological outcomes are comparable to open liver resection (OLR) [18]. There is a specific section involving HCC, stating that the available data from literature strongly suggested that LLR for HCC treatment is associated with reduced blood loss, transfusion rate, postoperative ascites, liver failure, and hospital stay with comparable operation time, disease-free margin, and recurrence rates [19,20]. Furthermore, in a propensity score-matched study focused on minor resections, a laparoscopic approach was found to be the only independent factor to reduce the complication rate in resections for HCC [21].

Thus, the adoption of LLR for HCC should now be recommended in each high-volume HPB center. During the initial phases, clinical practice should follow a step-wise approach, starting from minor liver resection in anterolateral segments, followed by major liver resections and resections of lesions located in posterosuperior segments, which are the most difficult due to the orientation of the transection planes. To guide this approach, several difficulty scores and classification systems have been described. Unfortunately, to date, most of the available scores consider only some aspects, and there is not a score able to predict all the different possible outcomes.

Even after the consensus statements, the role of LLR in some situations is still debated, such as for difficultly located HCC and for multiple or giant lesions.

## 3. Perioperative Management of HCC Patients Undergoing LLR

In the near-zero mortality era, with all technological advances available in surgical, anesthesiologic, radiological, and hepatological fields, attention must be focused not only on intraoperative aspects but also pre- and postoperative assessments. Meticulous preparation of the patient, with attention to every aspect concerning the different phases of management, is the key to finally overcoming different limits in the treatment of HCC patients.

### 3.1. Tumor Anatomical Modeling and Surgical Planning

One of the most important differences from other surgeries is that liver resections need a wide preoperative evaluation with very tailored surgical planning. A precise study of our patient’s anatomy, as well as the exact location of the lesion within the liver and its relationship to vascular structures, is essential to correctly plan LLR. Anatomical and positional aspects seem to be even more important in HCC surgery, given the importance of performing anatomical resections, which implies the removal of the entire portal territory nourishing the tumor, which could be associated with better short-term oncological results [14,22]. Therefore, different imaging modalities have been improved, such as 3D reconstructions for a more accurate study of exact lesion positioning with regard to vascular and biliary structures [23].

When dealing with large resections, especially in the case of cirrhotic liver, multiple tumors, or large-sized lesions, the risk for post-hepatectomy liver failure (PHLF) is still an important cause of mortality [24]. The preoperative evaluation should also be aimed to identify all the possible preoperative risk factors for PHLF in order to mitigate them and prevent fearsome postoperative complications as much as possible [25]. While the biggest part of these risk factors cannot be modified, we can act on the volume of the liver remnant. Thus, a precise volume analysis must always be carried out before a major hepatectomy. Vauthey et al. introduced a formula for a precise evaluation based on a correlation of liver volume with body surface area (eTLV: = −794.41 + 1267.28 × body surface area) [26]. As widely validated in literature, the estimated FRL can be calculated by the ratio of FRL volume and eTLV (FRL_Standard_ = LRV/TLV_Standard_ × 100%) [27]. It is considered safe an FRL of ≥20% of volume in case of normal liver, ≥30% after chemotherapy, 40% for steatotic and cholestatic liver, and ≥50% in case of cirrhosis [28].

### 3.2. Evaluation of Liver Function

Stratification in cirrhotic patients always has to be assessed using the Child–Turcot–Pugh (CTP) and the Model for End-Stage Liver Disease (MELD) score, as recommended in international guidelines [10,11]. In particular, the Barcelona Clinic Liver Cancer (BCLC) staging system is the most widely adopted, and it takes into account simultaneously the liver function evaluation with CTP score, patient performance status, and tumor characteristics, allowing a prognostic stratification and guiding treatment allocation [7,10]. However, both scoring systems present some weak points. They are not useful in non-cirrhotic patients, and they cannot accurately identify patients at risk for postoperative liver failure [29]. In particular, an impaired CTP can hide a wide range of cirrhosis severity (the “ceiling effect”), as well as a suitable CTP, cannot show different underlying conditions (“floor effect”) [30]. Similarly, newer scores have been proposed, such as ALBI and ABIC scores, but they cannot be used in all settings of patients, and they are not universally adopted [31]. Thus, other liver function tests should be used in addition.

The measurement of indocyanine green (ICG) clearance is widely performed in Asia, as well as in high-volume Western HPB centers. It is a dynamic method that evaluates the hepatic clearance of indocyanine green 15 min after its intravenous administration (ICG-R15), and it is usually delayed in cases of liver disease [32]. A decisional algorithm with excellent results in cirrhotic patients was developed by Makuuchi et al., according to which major resections should only be performed in patients with ICG-R15 lower than 10–20%, while neither minor resections when ICG-R15 is higher than 40% [33]. As a weak point, we can underline that it can evaluate only global liver function without specific information about the remnant. Furthermore, the use of ICG is impaired by hyperbilirubinemia since the uptake is mediated by common hepatic transporters.

Hepatobiliary scintigraphy with 99mTc-mebrofenin (HBS) is the most widely used nuclear medicine imaging technique to assess liver function. The 99mTc-mebrofenin extraction rate is correlated to underlying parenchymal status and to ICG clearance (sharing the same OATPB1/B3 transporters) [34]. In addition, it allows an evaluation of regional and segmental repartitions by the calculation of the 99mTc-mebrofenin extraction rate in the volume of interest, such as our FRL. At the segmental level, the FRL function appears to better predict the risk of PHLF than volumetric-based parameters [35]. Sadly, it is cost- and facilities-demanding, and it is impaired by hyperbilirubinemia.

### 3.3. Augmentation Volume Procedures

When needed, different strategies can be used to induce compensatory hypertrophy of FRL to reduce the risk of PHLF. To date, portal vein embolization (PVE) is considered the standard of care procedure for the FRL augmentation, with indications that include HCC on the cirrhotic liver. Up to 85% of patients can undergo liver resection after 4–6 weeks, which means that there is almost a 20% of failure rate due to both insufficient FLR or tumor progression in the waiting time [36]. To prevent tumor growth between PVE and LR, the addition of sequential preoperative trans-arterial chemoembolization (TACE) has been proposed, also obtaining a higher degree of hypertrophy [37]. However, the association of these procedures can lead to a worse inflammation of the hepatic pedicle, making the consequent surgical resection more difficult from the technical point of view.

Guiu et al. proposed the liver venous deprivation (LVD) technique, based on the simultaneous embolization of the hepatic vein (± the median) and the ipsilateral portal vein, with an amplatzer plug positioned at about 10 mm from the ostium [38]. Preliminary results from a study with HBS showed +66% FLR function at day 7 after LVD when compared with PVE, an increase in the kinetic growth factor of 75%, as well as encouraging perioperative and oncological outcomes [39]. Such results must be validated by further randomized studies, and its role in cirrhotic patients is debated [40].

Associated liver partition with portal vein ligation for stage hepatectomy (ALPPS) is a two-staged hepatectomy (TSH) firstly described by Schnitzbauer et al. in 2012, with the main advantage of sensitively reducing the delay among first and second procedures [41]. The main issue for ALPPS is an increased risk of postoperative morbidity and mortality, especially for cirrhotic patients. Actually, the best setting for ALPPS is represented by patients treated for colorectal liver metastasis with an age inferior to 60 years old, reaching a mortality among 5% [42], even if cases of successful ALPPS have also been reported for advanced HCC with portal vein thrombosis [43].

### 3.4. Evaluation of Portal Hypertension

According to different international guidelines, preoperative portal hypertension should be assessed in cirrhotic patients undergoing liver resections [10,44]. The gold-standard technique is HVPG measurement, but it is not routinely performed because of technical and logistical issues. Thus, other methods have been proposed. In cirrhotic patients, a liver stiffness of <20 kPa and a platelet count of >150,000/dL are associated with a very low risk of clinically significant portal hypertension (CSPH) [45]. Recently, spleen stiffness has been suggested as a new non-invasive tool to predict CSPH and post-surgical morbidity and mortality [46]. Even if very promising, further prospective studies are needed before its routine use in the treatment algorithm for cirrhotic patients.

Furthermore, even if liver resections in patients with portal hypertension have traditionally shown high morbidity, thanks to ongoing improvements in surgical technique and intensive care management, they are considered feasible in selected patients in high-volume centers, leading to suitable long-term results, especially after LLR [47,48]. Azoulay et al. showed 79 patients with acceptable mortality, morbidity, and liver decompensation rates for HCC patients with CSPH. Furthermore, the laparoscopic approach was the sole predictor of a textbook outcome [49].

### 3.5. Intraoperative Management

Thanks to developments in surgical and anesthetic techniques, high-volume centers have reported operative mortality for LLR of less than 5% [50]. However, LLR for HCC has to face specific risks that must be well managed by both the surgeon and anesthetist by using a standardized perioperative protocol.

LLR carries an important risk of blood loss for the dissection of the hepatic vein, inferior vein cava, portal vein, and the transection of a highly vascularized parenchyma. Increased blood loss and perioperative blood transfusions are associated with worse perioperative morbidity and mortality [51]. Both surgeons and anesthesiologists must contribute to decreasing intraoperative blood loss. Surgeons can use vascular clamps intraoperatively, with more or less important hemodynamic implications: even if some small resections on a normal liver can be avoided, in order to not cause an ischemic injury to the remaining liver and intestinal congestion, a Pringle maneuver (selective or not) should be prepared when performing major resections, as recently shown in a propensity-matched analysis on 209 patients [52]. Anesthesiologists can reduce central venous pressure (CVP) during the parenchymal transection: randomized trials have shown how a low CVP during parenchymal transection results in decreased blood loss and transfusion requirements [53]. A problem with this procedure could be the possibility of renal injury, especially in cirrhotic patients, but to date, there is no evidence of such improved risk.

Air embolism is another known intraoperative complication of LLR that can result from the use of pneumoperitoneum, with various precautions that have been proposed to reduce this risk. However, more recent evidence showed no increased risk of air embolism during LLR compared with OLR, neither depending on patient positioning [53].

## 4. Overcoming old limits

### 4.1. LLR in Cirrhotic Liver

HCC develops in a cirrhotic liver in approximately 80–90% of cases, and the incidence of cirrhosis is expected to increase worldwide due to the prevalence of obesity, fatty liver disease, and alcoholic steato-hepatitis [54]. After an initial phase in which the presence of cirrhosis was considered a contraindication to laparoscopy, several studies investigated the outcomes of LLR in cirrhotic patients. These patients often present a risk of postoperative hepatic decompensation and failure, as well as low platelets and impaired coagulation. A metanalysis of 11 studies comprising 1618 patients indicated a 16–26% reduction in the hazard ratio of death for patients with HCC and cirrhosis who underwent LLR [55]. In addition, LLR was associated with reduced blood loss, reduced major complications, and shorter length of hospital stay.

When considering liver resection in patients with liver cirrhosis, it is important to consider not only the oncological outcomes but also the surgical stress on both the patient and the liver. An important consequent advantage of LLR in cirrhotic patients is the lower incidence of postoperative liver failure and ascites, given the reduced interruption of portosystemic shunts and the avoidance of electrolyte imbalances as a result of the exposure of the abdominal content to the air [12]. Furthermore, the reduced invasiveness of laparoscopy, which minimizes liver manipulation, preserves collateral vessels and the abdominal musculature, and can be a key factor in expanding the classic limitations of liver surgery [56]. In fact, the advantages of LLR have been confirmed even in advanced Child-B cirrhotic patients: a propensity score-matched study involving international high-volume centers showed reduced blood loss, morbidity, and major complications in the LLR group in this setting [57].

### 4.2. Giant Tumors

In the first years of the spread of LLR, huge-volume tumors were considered a contraindication due to both technical and oncological issues. The last decades saw an extension of tumor burden-related indications for LR. Currently, the EASL guidelines recommend LR in cases of a resectable lesion regardless of its size, while the AASLD guidelines advocate LR in patients with Child–Pugh A cirrhosis and resectable HCC with a diameter less than 5 cm. Meanwhile, according to APASL, all tumors without extrahepatic metastases are potentially resectable regardless number and size of lesions [10,11,44]. However, expert centers published several experiences involving LLR for giant tumors, and, as the technical challenges become easier to face with widespread minimally invasive experience, the fear for oncological results and PHLF still exists.

Recently, Hong et al. published suitable long-term outcomes from a nationwide cohort of 466 patients with large HCC, suggesting a worse prognosis in subgroups with low platelets and tumors >10 cm [58]. Similarly, suitable long-term outcomes for giant HCC were shown by Thng et al., who found the presence of satellite nodules and blood transfusions as the only negative predictors of worse prognosis [59]. The safety of LLR for large malignant tumors was previously reported by different authors, with a recent international multicenter matched cohort study with regression discontinuity analyses that also concluded that the safety of MILS also for tumors larger than 10 cm, even if technically demanding [60,61]. Accordingly, the size of the tumor is taken into account in the Iwate difficulty score so that tumors larger than 3 cm are considered of increased difficulty [62].

Giant tumors indeed are extremely demanding to operate on, where limits of what is considered technically feasible can easily be reached. The placement of trocars, the mobilization of a liver lobe, and the accidental tumor perforation by shear forces are examples of possible intraoperative difficulties. Further arguments against the laparoscopic procedure for giant tumors include that conventional recovery bags are too small, and a comparatively long incision is required to retrieve the specimen. In this respect, the question of feasibility depends primarily on whether the resection can be performed safely, and, if technically feasible, well-known advantages of LLR have also been confirmed in the literature for giant tumors [63].

Therefore, the decision whether to operate laparoscopically or rather conventionally open should be based on the findings and, again, on the own learning curve and personal experiences made. In our experience, the trocars’ positioning is fundamental, and it depends on both the experience of the surgeon of the center, as well as on the segment to be resected. The manipulation of the liver must be performed with caution, using protections under the hepatic retractors so as not to damage the tumor capsule. The mobilization of the liver can also be performed laparoscopically in the case of large tumors, in which case the correct trocar positioning and the experience of both the surgeon and the operator that holds the rotating camera are once again important.

### 4.3. Multiple Tumors

Besides giant tumors, the role of LLR in the case of multiple HCCs is also debated. From an oncological point of view, in Western countries, the best candidates have always been defined as those with a single tumor, and the treatment of multifocal non-metastatic HCC consisted of LT, within Milan criteria, or ablation/chemoembolization for the remaining patients [10]. As early as 2014, Eastern countries did not consider the presence of multiple HCCs as a contraindication, and a recent Japanese national series reported better results in Child A patients than radiofrequency or TACE in terms of OS, albeit at the cost of greater morbidity [64]. A definitive green light came from a randomized trial confirming that LR provided better OS for patients with multiple HCC outside of Milan criteria than TACE [65], even if the number of tumors was an independent risk factor. Recently a propensity score-matched analysis including multiple HCC within Milan criteria finally confirmed the safety of the laparoscopic approach [66].

Obviously, published data come from the long experience of high-volume centers for complex LLR. In our experience, an expert ultrasonography-guided parenchymal dissection is indispensable [67]. Three-dimensional laparoscopy should be mentioned as an additional supportive visual tool, as well as the use of ICG that can further help to both detect superficial lesions and guide difficult parenchymal dissection [32]. Further technological research is supposed to help surgeons in this scenario, such as the application of virtual realities, which could also be beneficial in this context [68].

In conclusion, LLR should be considered in multinodular HCCs, but more robust studies are needed to support clinical practice. A personalized strategy can also be proposed, combining both laparoscopic ablation and resection when technically demanding, and the size and position of the lesions can benefit from it [69].

### 4.4. Difficult Positions

**Figure 1:**
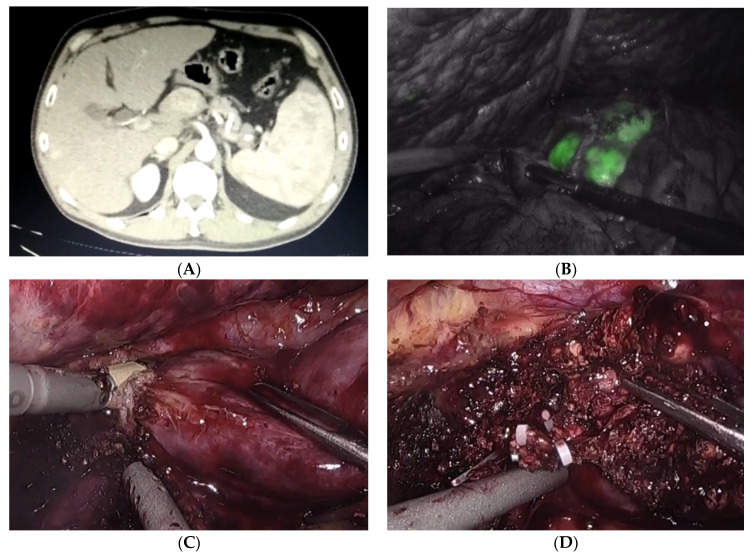
Laparoscopic resection of caudate lobe for hepatocellular carcinoma. (**A**) CT scan with arterial wash-in. (**B**) ICG enhancement of the lesion, assuring negative resection margins. (**C**) Parenchymal transection. (**D**) Securing spigelian vessels.

**Figure 2:**
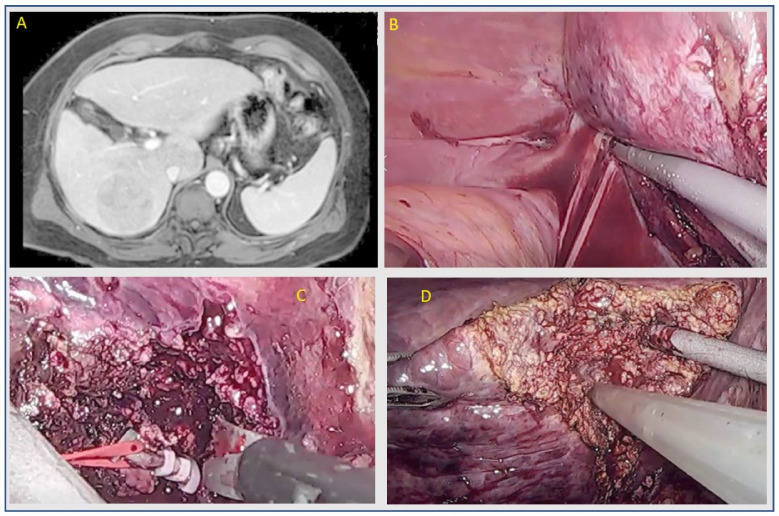
Laparoscopic right posterior sectionectomy. (**A**) CT scan with portal wash-out. (**B**) Mobilization of right lobe. (**C**) Selective ligation of right posterior portal branch. (**D**) Parenchymal transection by using ultrasonic cavitron.

The technical difficulty associated with LLR is linked to different aspects, such as parenchymal transection, hemostasis at the transection plane, and limited ability to explore deep and posterior regions of the liver. Therefore, LLR was initially reserved for superficial or left-sided lesions. The successive improvements in laparoscopic techniques and the introduction of new technologies mean that LLR is technically feasible in postero-superior (PS) segments (I, IVa, VII, VIII), too [70] (Figure 1 and Figure 2). LLR of PS segments are considered major liver resections, according to the most recent international consensus [18], since they have shown a significantly longer operative time, length of hospital stay, rate of open conversion, and estimated blood loss when compared to antero-lateral resections [71].

At the same time, improved laparoscopic techniques, better visualization of the operative field using a flexible laparoscope, and routine use of an ultrasonic cavitron for transecting the deeper portion of the liver parenchyma have allowed to reach excellent outcomes for LLR for HCC located in the PS segments, resulting in reduced blood loss, fewer complications, and shorter postoperative hospital stay compared with OLR tor the same segments in retrospective studies [72,73]. An international multicenter randomized trial for LLR in PS segments (orange segments trial) is still ongoing, and it will allow obtaining a definitive confirmation.

Segments 7 and 8 are the more posterior ones, rated as 5 on the Iwate score, because of unfavorable working angles and a poor operating view, especially with the classic trocar positioning, from caudal to cranial. Thus, different approaches have been proposed: Morise proposed a left lateral position for posterior sectionectomy and the semi-prone position S7 segmentectomy, with or without an intercostal placement or a lateral positioning of the trocars [74]. Newer dissection strategies have also been proposed, such as the diamond technique, allowing safe LLR in PS segments, even in cirrhotic patients [75].

In our experience, it is the surgeon’s ability to master both hepatic anatomy and laparoscopic liver surgery that makes the difference, with the need to know how to deal with any dangerous bleeding from the hepatic veins and probably know when to convert to prevent them happen. The approaches described in the literature can probably all be used indifferently but consistently with the experience of the surgeon and the center. Finally, also for the resections of the PS segments, and perhaps above all, technology can once again come to our aid for the resections of patients with HCC. In fact, the use of the ICG allows obtaining both a positive and a negative counter-staining, facilitating the transection line in the Glissonian approach for anatomical resections, as originally described by Takasaki, useful specifically for HCC treatment [32,76]. At the same time, 3D modeling or virtual reality could help to clarify the difficult relationships of the lesions with the hepatic veins, even if their real role needs to be proved [77].

### 4.5. Repeat Resection

As already mentioned, HCC has a high risk of recurrence after both LT and liver resection. Thus, repeat laparoscopic liver resection (RLLR) has increased thanks to the progressive wide adoption of LLR. Furthermore, LLR reduces the risk of further adhesions. Kanazawa et al. showed that the operation time for RLLR after previous LLR was significantly shorter than after OLR [78]. Belli et al. reported fewer postoperative complications, lower bleedings, and shorter hospital stay after RLLR than repeat OLR [79]. Recently, the feasibility of a laparoscopic approach for repeat resection after LT was also reported, pushing the limits of MILS even further [80].

Finally, Morise et al. recently published an international multi-institutional propensity score-based study of RLLR for HCC, showing less blood loss and hospital stay for the laparoscopic group, even if the LLR was preferred for patients with favorable tumor characteristics [81].

### 4.6. Robotic Liver Resection

Since the first series of robotic liver resections (RLR) reported by Giulianotti et al. in 2003, different advantages of this approach have been proposed: from the ability to articulate the instruments and the magnified three-dimensional vision to ergonomic advantages for the surgeon [82,83]. Several studies have investigated the safety and effectiveness of RLR in different situations, leading to the first international consensus statement on RLR in 2018 [84,85].

Safe and effective RLR for tumors located in the PS segment has been reported by several authors [86]. Similarly, robotic hemi-hepatectomies were associated with less intraoperative blood loss and a shorter operation time than LLR. Hu et al. published a meta-analysis including 487 RLR and 902 LLR showing fewer bleedings for RLR, with longer operation time than LLR [87]. There was no significant difference in hospital stay, conversion rate, R0 resection rate, and total complication rate between the two groups.

The high cost of treatment, as well as logistic and organizational aspects, may be the biggest shortcomings in the development of robotic surgery.

### 4.7. MILS and Liver Transplantation

Since the first laparoscopic hepatectomy (LDH) for a living donor LT (LDLT) was performed for a pediatric recipient by Cherqui in 2002, many transplant centers worldwide have adopted this approach, even if some concerns about donor safety still exist [88]. Hong et al. in 2021 published results from a Korean multicenter study on more than 500 LDH, showing similar outcomes to the open approach in terms of safety, with a decreasing operation time [89]. Recently, two meta-analyses involving more than 1000 patients concluded the safety of LDH while showing some advantages in terms of lower blood loss and shorter hospital stay [90,91].

Similarly, the first robotic donor hepatectomy was a right hepatectomy reported by Giulianotti et al. in 2012, with the aim of applying the supposed advantages of the robotic approach also in the field of LT [92]. The first series published by Chen et al. showed comparable results for complication rates, blood loss, and recovery of donor liver function when compared to open hepatectomy, with a shorter length of stay and less postoperative pain, without open conversions; the robotic group had longer operation time [93]. Recent systematic reviews support the safety of the robotic approach, suggesting technical advantages regarding hilar dissection, with no major difference in terms of ischemic time or cosmesis [94]. Therefore, robotic donor hepatectomy has been proposed as a viable option for experienced surgeons in the latest recommendations on robotic liver surgery [85].

Finally, what seemed to be the major limitation for laparoscopic liver surgery, namely the implantation of the liver graft in the recipient, was also overcome in 2021, a historic event for LT. The operation carried out by Suh et coll. lasted 960 min, including pure laparoscopic total hepatectomy and pure laparoscopic implantation, through a suprapubic incision, and was shown to be safe, without postoperative complications [95]. Further prospective studies on larger sample sizes will have the task of clarifying the benefits of such an incredible procedure.

## 5. Conclusions

In conclusion, with the advances in surgical techniques and perioperative management experienced in the recent two decades, indications for LLR in HCC patients have tremendously improved and become technically practicable for the biggest part of lesions. Current advances in both surgical and medical treatment for HCC will probably redefine the actual frontiers of LLR by including patients with more advanced disease. With the exponential growth of LLRs performed around the world, it is important to know the specific limits of this approach in HCC patients in order to put into practice all the pre-, intra-, and postoperative precautions to overcome them, making this technique the standard of care within high-volume hepatobiliary centers. Expertise and learning curve should remain the mainstay, and the selection of appropriate candidates with meticulous preparation are the key points to ensure the success of the approach.
